# Adenoid cystic carcinoma of the breast - Discordant size on imaging and pathology: A case report and review of literature

**DOI:** 10.1016/j.amsu.2019.04.007

**Published:** 2019-05-06

**Authors:** Slava Agafonoff, Anna Sobolewski, Timothy S. Braverman

**Affiliations:** Jewish Hospital, Cincinnati, OH, USA

**Keywords:** Adenoid cystic carcinoma, Breast, Sentinel lymph node biopsy, MRI, Case report

## Abstract

**Introduction:**

Adenoid cystic carcinoma (ACC) is an uncommon tumour of the breast. It is known for its rare lymph node involvement and distant metastasis. A triple-negative breast cancer that has a favorable prognosis compared to other triple negative ductal carcinomas, it accounts for approximately 0.1–1% of all breast cancers.

**Presentation of case:**

We report a case of a 69-year-old female with a palpable left breast mass who underwent multiple imaging modalities with significant size variance between the studies. Breast conserving therapy (BCT) was performed with axillary sentinel lymph node biopsy (SLNB) followed by radiation therapy (RT). Pathological examination confirmed the tumour as ACC.

**Discussion:**

ACC, known as an persistent if low-grade malignant tumour of salivary gland, is considered to have low-malignant potential in the breast. It is a very rare subtype and from this scant data, there is minimal mention about size discrepancy between imaging modalities such as ultrasound and MRI.

No consistent MRI features have been demonstrated, with the exception of T2 hyperintensity in larger lesions and T2 iso-intensity in smaller lesions. Ultrasound demonstrates primarily a hypoechoic or heterogenous mass with minimum vascularity, consistent with our radiographic findings.

**Conclusion:**

ACC is a rare entity in breast cancer pathology. Its size can be highly variable as measured by various radiographic modalities, and final Pathology from the surgical specimen is, as always, required for an accurate tumoral diameter. With that caveat, careful utilization of pre-operative imaging modalities is critical in pre-surgical planning to choose the appropriate surgery.

## Highlights

• MRI can help in pre-operative planning.
• High-grade/anaplastic-appearing tumours may have a more aggressive clinical course.
• Discrepancy between imaging size and final pathology can be present and affect surgical management.
• Breast conserving therapy with adjuvant radiation therapy improves survival.

## 1 Introduction

ACC accounts for 0.1–1% of all breast cancers [4,5]. The major issue in the management is, due to its rarity, the absence of clinical trials enrolling these patients to determine the best treatment plan. Again, given its rarity, management and diagnostic imaging recommendations have not been established. It typically presents in Caucasian women in their 6–7th decade as a palpable and often tender mass. It is usually a “triple negative” (ER, PR, and HER-2) breast cancer, precluding hormonally-targeted therapy [3].

Here, we report our institutional experience with ACC of the breast with the aim of providing additional current literature and our management.

This case report was reported in accordance with the SCARE criteria [11].

## 2 Presentation of case

**Fig. 1:**
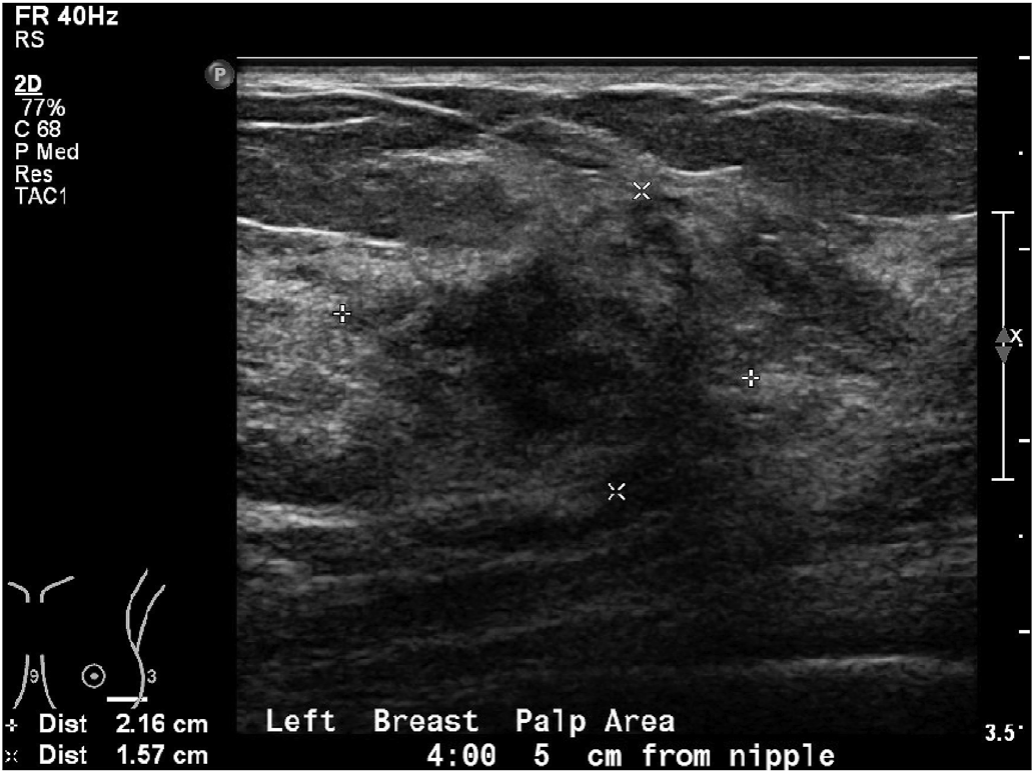
Ultrasound left breast the region of palpable abnormality shows an area of heterogeneous hypoechoic tissue at approximate 4:00 o'clock, 5 cm from the nipple measuring up to 2.2 × 1.6 cm.

**Fig. 2:**
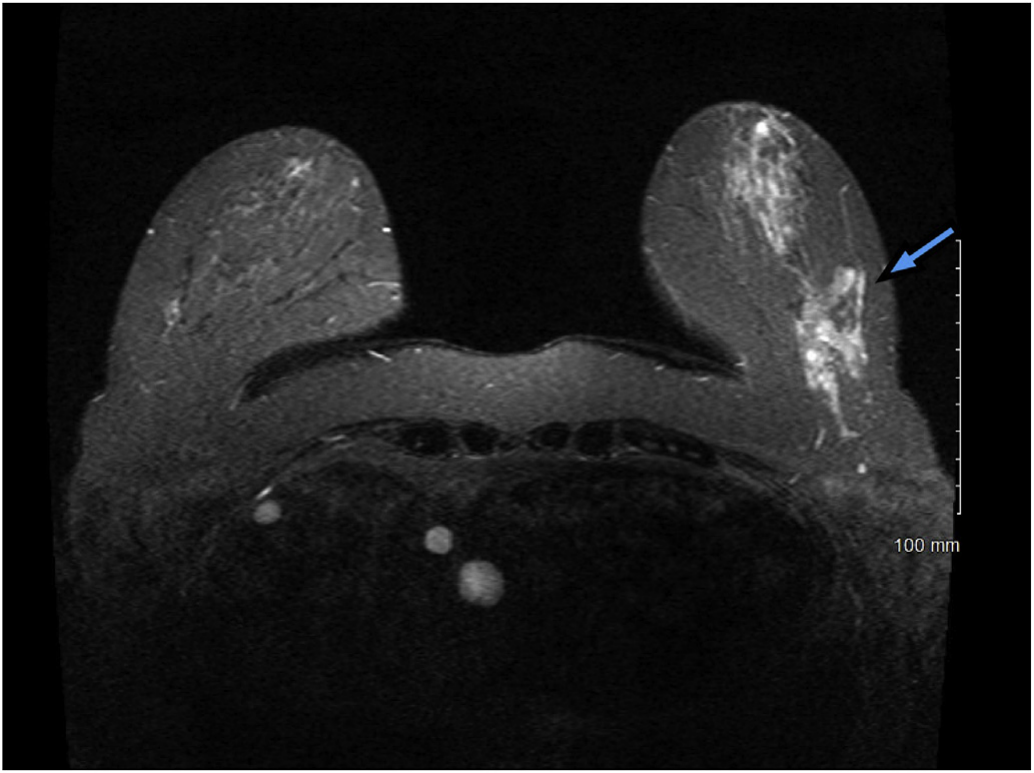
MRI of Left breast demonstrates mild background parenchymal enhancement, Mass enhancement within the lower outer aspect of the left breast which measures 5 cm in AP dimension 3 cm transverse dimension and 2 cm in craniocaudal dimension corresponding to area biopsied.

This 69-year-old female, with a past medical history of thyroid disease and asthma, presented with a tender, palpable, lower outer quadrant breast mass. Family history is positive for conventional breast carcinoma in the mother. The patient noted the mass approximately a week prior to her initial visit. She had prior mammograms 6 months and one year prior to her presentation, both negative. Ultrasound (Fig. 1) demonstrated a 2.2 cm heterogeneous, hypoechoic tissue mass at 4:00, and 5 cm from the nipple. Core biopsy was diagnostic of ACC, with negative ER, PR, and HER-2. MRI (Fig. 2) was obtained due to very dense breast tissue, particularly with negative current mammogram, revealing a 5 × 3 × 2 cm mass. On physical exam, a 1 cm tender palpable mobile mass was identified, with local ecchymosis. No other masses or skin changes are found, and there was no palpable axillary lymphadenopathy. After discussion with the patient, a left partial mastectomy was performed, with left axillary SLNB. An additional margin was obtained to achieve complete resection. BioZorb was placed for post-operative radiation localization.

Our pathology confirmed ACC, with mass lesion measuring 35 mm in greatest extent within the primary excision. The SLNB was negative (0/1). The pathologic stage on the synoptic report was pT2N0 and the tumour grade 2. As is the usual case with ACC, no in-situ carcinoma was found.

No loco-regional recurrence or distant metastasis was found at 6 month follow-up. Repeat mammogram at 6 months demonstrated a Bi-RADs-3. MRI will be repeated at the 1 year follow-up.

## 3 Discussion

ACC was first described by Billroth in 1856 and called ‘cylindroma’. Much later, further scientific investigation by Geschickter and Copeland discovered its eccrine gland origin in 1945 [1,2]. ACC is generally negative for estrogen (ER), progesterone receptors (PR), and HER2. ACC is known of primarily as a persistent if low-grade malignant tumour of major salivary glands, however, and is considered to have low malignant potential in the breast. It is a very rare subtype and from this scant data, there is minimal mention about size discrepancy between imaging modalities such as U/S and MRI. Yan et al., presented a case in which their imaging demonstrated a clinical breast of 1.5 cm, ultrasound diameter of 2 cm, without MRI, the final pathological malignancy 5.5 cm in diameter. The patient underwent a simple mastectomy even though she was a candidate for BCT, serendipitously the appropriate therapeutic approach [6]. Our MRI demonstrated a 5.5 cm lesion and ultrasound diameter of 2.5 cm with final pathology of 3.5 cm. In our case, BCT with radiotherapy worked extremely well.

**Fig. 3:**
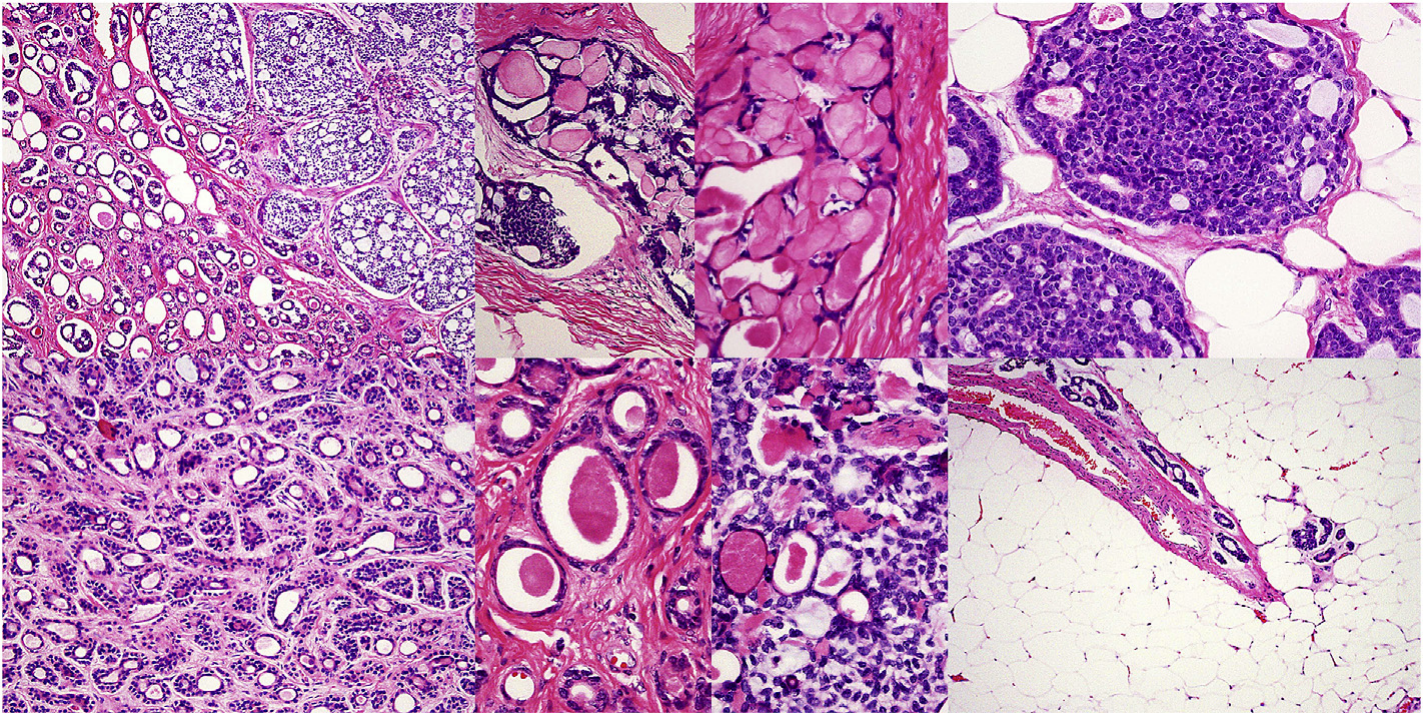
Excision (less than total mastectomy) with numerous tumour patterns, primarily gland-like to cribriform, some areas dominated by basement membrane spherules, others by dense myoepithelium, the bottom right hand corner with distantly and inconspicuously infiltrating tumour at margins.

**Fig. 4:**
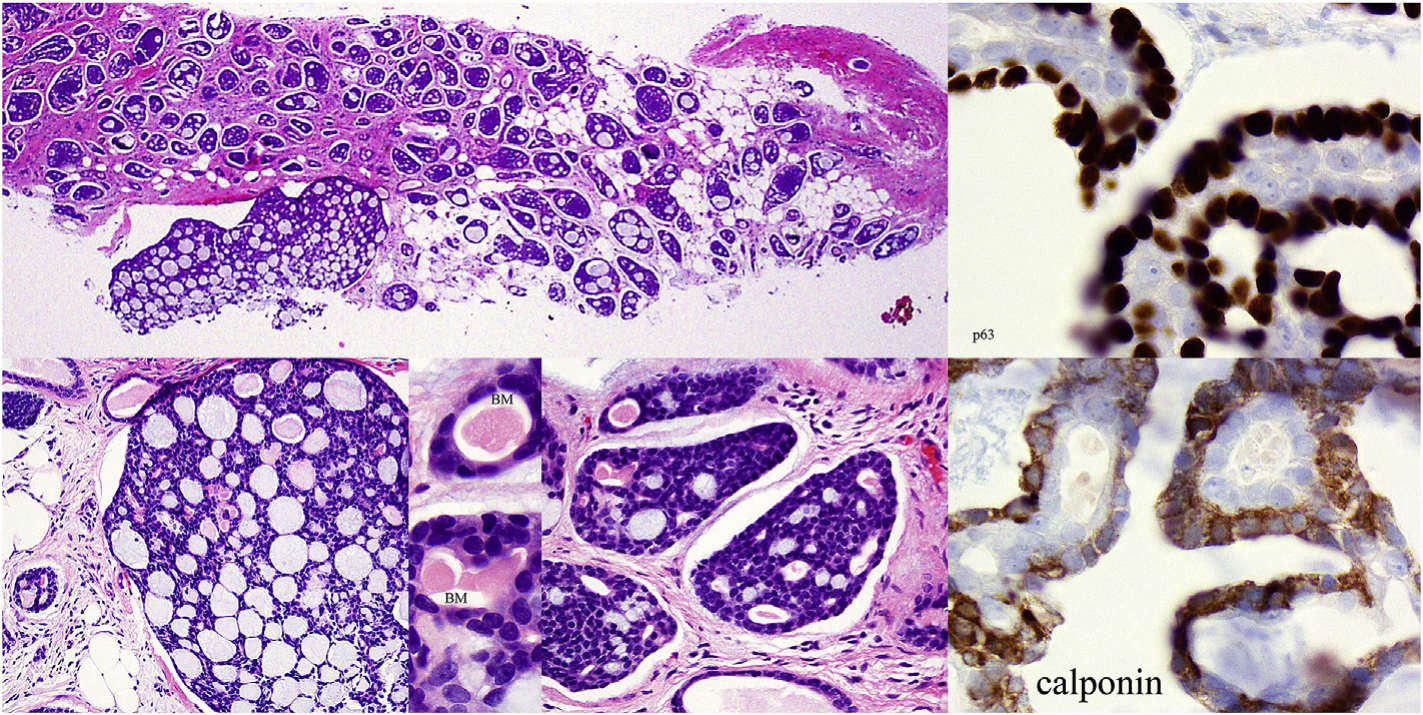
Core biopsy with variable histologic pattern, p63 (nuclear) and calponin (cytoplasmic) immunostains highlighting the positively staining myoepithelium at the periphery of the negatively staining epithelium.

As the breast is developmentally a modified sweat gland, and close analogy exists between sweat gland and salivary gland tumours, some neoplasms of the breast resemble those seen in the latter organs. This includes ACC, an important if rare inclusion. Differentiating itself from other morphologically similar neoplasms and conditions (e.g. collagenous spherulosis), ACC shows, as in the salivary glands, an epithelial/myoepithelial proliferation with two types of cavities, 1) true glands, and 2) gland-like spaces containing dense, eosinophilic basement membrane material and basophilic mucin, characteristic of this neoplasm (Fig. 3). If nerves are present in the specimen, perineural invasion, a common feature of ACC in the salivary glands, may be present, important evidence of the diagnosis. As is typically present, and shown by this case, multiple patterns of epithelial and myoepithelial proliferation are present, ranging from simple gland-like formations to cribriform architecture, to solid nests of cells. In all cases of ACC, myoepithelium (highlighted by several immunohistochemical stains, including those documented here) accompanies the true epithelium, a unique finding for a malignant tumour, even a primarily low-grade if persistent one such as ACC (Fig. 4).

Additional possible findings include the following: A distinctive t(6; 9) genetic translocation results in MYB-FIB fusion, activating the MYB oncogene (with better data from the more common salivary gland tumour, where the fusion is common). Axillary nodal metastases are extremely rare, but some patients develop local recurrence or pulmonary metastases, including temporally distant from initial diagnosis/therapy. Nonetheless, the prognosis for the overall tumour group remains quite good. The relationship between microscopic grading and prognosis remains controversial, some authors offering that high-grade/anaplastic-appearing tumours may have a more aggressive clinical course.

On mammograms, the majority of ACC's of the breast present as high-density masses with irregular or lobulated shape and indistinct margins, described as malignant-appearing, in contradistinction to our case [2]. The mammogram in our patient was noted to be BiRAD-2 without direct or indirect signs of malignancy, likely in part due to the density of the patient's breast tissue. Given the dense breast tissue and lack of mammogram findings, we obtained an MRI.

No consistent MRI features have been demonstrated with the exception of T2 hyperintensity in larger lesions and T2 isointensity in smaller lesions. U/S usually demonstrates hypoechoic or heterogenous mass with minimum vascularity, consistent with our radiographic findings [3,7].

Our MRI demonstrated a 5 cm lesion which would be considered a stage IIB (locally advanced breast cancer) but our final pathological specimen was 3.5 cm, therefore, stage IIA disease. Without no established guidelines, this appreciable discrepancy may have affected our pre-operative planning and the choice of breast surgery, a significant point of interest in this malignancy. Consider that, were the patient to have positive margins given the discrepancy, a second operation would be required to complete the excision, either additional BCT, or mastectomy. It would be useful if the extent of the ACC could be determined more accurately radiologically. As MRI has greater sensitivity than mammogram and U/S in determining the true tumour extension, in cases with core biopsy-established diagnosis of ACC, this should be a requirement for treatment planning. This information demonstrates a fast growing lesion with discordant findings on mammogram, ultrasound, MRI and final pathology.

Treitl et al. state local excision is recommended for grade 1, simple mastectomy for grade 2 and mastectomy with axillary dissection for grade 3 tumours, however, tumour grading in ACC is difficult even on resections, most tumours behaving in a persistent if low-grade fashion. In our case, treatment with BCT and radiation has shown no recurrence to-date [3].

Metastasis from ACC is uncommon, the lungs the most common site, with liver, bones and kidney less likely. Lymph node involvement is very rare (not exceeding 2%), however, SLNB is required for ACC, as with any invasive breast primary. In a retrospective case series from with follow up of 20 years, patients with pure ACC (n = 20), out of a 15,749 total cases of invasive breast cancer, 15 of those had SLNB with pathologically node-negative lymph nodes, and no evidence of any lymph node involvement in 20 year follow up [8,9].

There is no indication for adjuvant chemotherapy and several small studies suggest no survival benefit on overall survival [9].

There is improved survival with adjuvant radiation therapy. Patients receiving lumpectomy and radiation therapy had a survival benefit of 9% at 5 years and 21% at 10 years. Therefore, radiotherapy decreases local recurrence for patients who had undergone BCT like in our case [3,6,8,10].

**Fig. 5:**
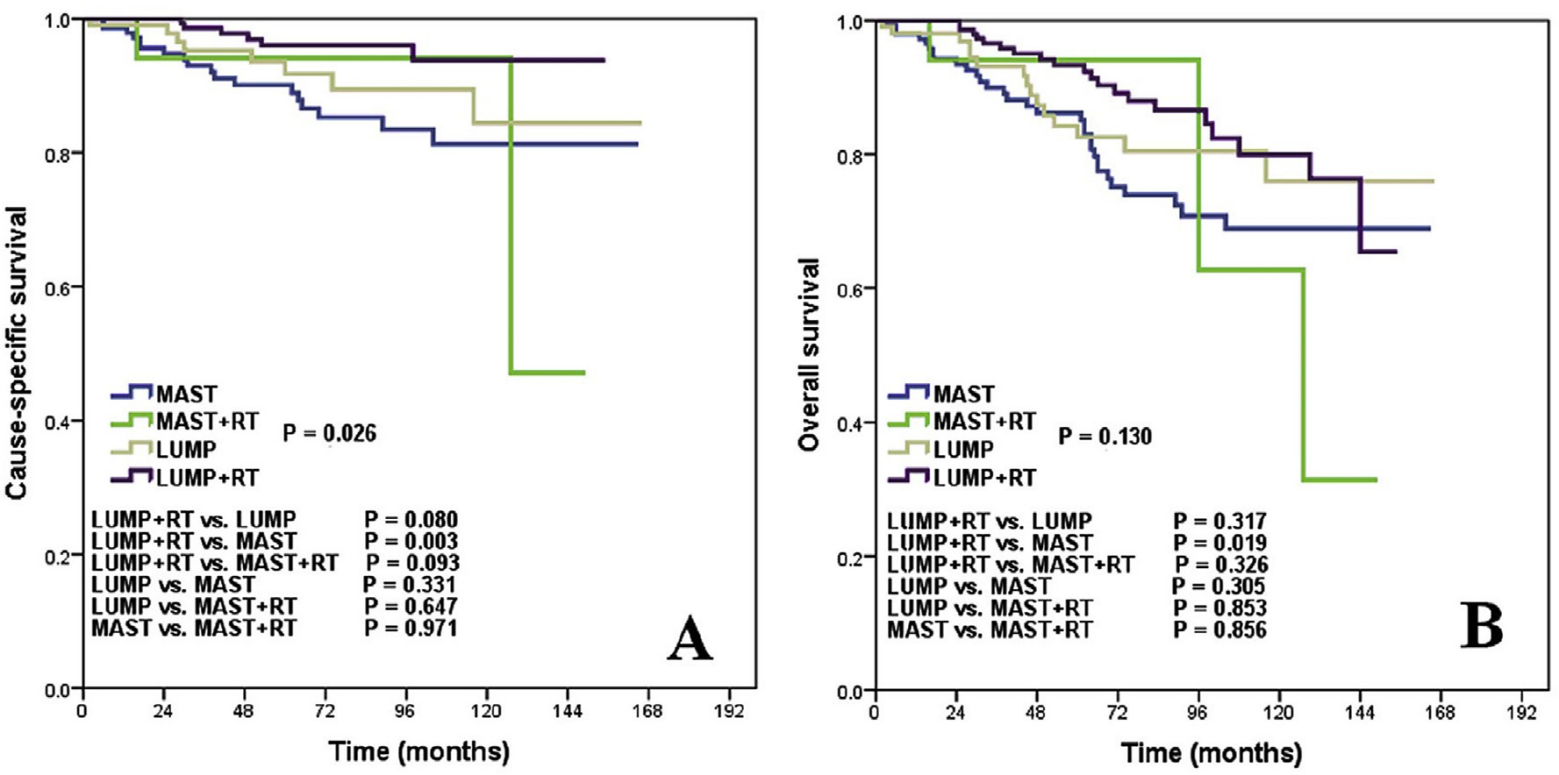
Kaplan-Meier survival curves showing a comparison of (A) cause-specific survival and (B) overall survival among LUMP, LUMP þ RT, MAST, and MAST þ RT patients.

Sun et al., presented 478 patients who underwent excisional biopsy (“lumpectomy”) (107) vs. excision and radiotherapy (197). The second group demonstrated better survival advantage with P = 0.018. Their results suggest that BCS was the optimal treatment of choice for patients with ACC of the breast, and that adjuvant RT could further improve the survival rate. Recurrence rate after mastectomy is lower than BCT, therefore mastectomy with clear margins may be preferable to excision. Though recurrence and survival benefits are gained by mastectomy over excisional biopsy plus radiotherapy, the 5-year loco-regional control rate was 100% and 93% in the mastectomy and BCS groups, respectively (p = 0.16) [10]. (Fig. 5). Even though our patient only had a 6 month follow up which is not adequate time length for recurrence, it is reassuring she had no signs of disease. At one year, we will obtain an MRI to assess further and likely yearly after that for continued monitoring.

## 4 Conclusion

In conclusion, ACC is a rare entity in breast cancer pathology. Its size can be highly variable between radiologic methodologies, and final pathology from the surgical specimen is required for an accurate tumoral diameter. Noting all of the above, there remains no consensus on the optimal treatment for ACC, in part due to its rarity. Careful utilization of pre-operative imaging modalities is critical in pre-surgical planning to choose the appropriate surgery.

## Ethical approval

Ethical approval is exempted as this is a case report.

## Sources of funding

No source of funding

## Author contribution

All authors contributed to the writing of this paper.

## Conflicts of interest

There are no conflicts of interest.

## Research registration studies

Not applicable.

## Guarantor

Guarantor is Dr. Slava Agafonoff.

## Patient consent

Written/Verbal informed consent was obtained from the patient for publication of this case report and accompanying images. A copy of the written consent is available for review by the Editor-in-Chief of this journal on request.

## Provenance and peer review

Not commissioned, externally peer reviewed.
